# The Role of Clinical Psychology and Peer to Peer Support in the Management of Chronic Medical Conditions – A Practical Example With Adults With Congenital Heart Disease

**DOI:** 10.3389/fpsyg.2018.00731

**Published:** 2018-05-30

**Authors:** Edward Callus, Gabriella Pravettoni

**Affiliations:** ^1^Clinical Psychology Service, IRCCS Policlinico San Donato, San Donato Milanese, Italy; ^2^Applied Research Division for Cognitive and Psychological Science, Istituto Europeo di Oncologia s.r.l., Milan, Italy; ^3^Department of Oncology and Hemato-Oncology, University of Milan, Milan, Italy

**Keywords:** clinical psychology, peer to peer support, non-profit patient associations, congenital heart disease, psychosocial, chronic illnesses, treatment preferences

## Abstract

Clinical psychology services and peer to peer support can both contribute in increasing the psychological wellbeing of patients with chronic medical conditions. In this perspective paper, indications are given about the provision these services for the specific case of adults with congenital heart disease. These patients are at an increased risk of psychological distress, neurocognitive deficits, and social challenges. The psychosocial characteristics and mental health treatment preferences of these patients are briefly described, followed by guidelines and indications for the implementation of clinical psychology services. The most structured peer to peer program available for this population is subsequently illustrated and finally, specific benefits and challenges when it comes to the integration of both services are reported.

## Introduction

There are often difficulties in health services when it comes to financial sustainability, and patients’ associations are created to attempt to provide welfare services which would be difficult to obtain otherwise (Geier, 2015). Only the patients have disease specific skills and knowledge based on their experience, and a desire to exert control over their health condition, which can be a great resource (Pravettoni et al., 2016).

It is therefore important to enable the patients to feel empowered, and this can only be achieved if they perceive that they have control on their condition (Kondylakis et al., 2012). It is also important to create systems in which as many psychosocial elements as possible are taken into consideration, in order to create a personalized approach in healthcare, which improve and facilitate the communication between the physician and the patient (Kondylakis et al., 2013).

For this reason, it would be ideal to provide multidisciplinary family-centered psychosocial care in medical settings, such as pediatric cardiology and adult congenital cardiology units. This goal can be achieved by including psychologists, social workers, and clinical nurse specialists to care for patients with congenital heart disease (CHD) and their families, who have specific training when it comes to this population (Utens et al., 2017). Psychocardiology, a branch of clinical psychology, is an effort to see how psychology can contribute in the prevention, treatment, and rehabilitation of patients with cardiac disease (Molinari et al., 2006; Callus et al., 2010).

Peer to peer support can be an occasion to bolster patient resources, and to decrease a sense of isolation. This has been reported clearly by the World Health Organization, in which the strengthening of social relationships is identified as a health promotion strategy, endorsing initiatives which increase supportive resources such as mutual aid (World Health Organization, 2009).

There is a lot of variation in the description of peer interventions, but all seem to include emotional, informational, and appraisal support in some combination. Apart from there being positive outcomes, there could be adverse ones, such as exploitation and overburdening of peers and their inappropriate replacement of professional services (Dennis, 2003).

## Congenital Heart Disease: Characteristics and Psychosocial Needs

Congenital heart disease is the most common inborn defect with an approximate prevalence of 9.3 newborns for every 1000 births (van der Linde et al., 2011). Thanks to the advances and triumphs of cardiovascular medicine and surgery in the 20th century most of these patients survive to reach adulthood, causing a constant increase in the number of adults with CHD (ACHD) (Marelli et al., 2007; Moons et al., 2010) which have reached a total of 60% of the CHD population (Marelli et al., 2014).

More than a third of the patients is born with “critical heart disease” involving life threatening malformations which necessitate palliative or corrective surgery in early life and ongoing medical care (Samanek, 2000; Marino et al., 2001; Gatzoulis et al., 2005) and they are confronted with lifelong cardiac and non-cardiac comorbidities and challenges (Baumgartner et al., 2010).

Children with CHD have more difficulties when it comes to the behavioral, emotional, and neuropsychological aspects, in comparison to their healthy peers (Karsdorp et al., 2007; Bellinger and Newburger, 2010; Marino et al., 2012). In some cases, this can give rise to parental overprotection, even though this behavior was not reported to be indicative of all or even most parents (Verstappen et al., 2006).

When it comes to ACHD, they are at an increased risk of psychological distress, neurocognitive deficits, and social challenges (Lui et al., 2017). Three studies conducted in North America which also included clinical interviews reported that one-third of ACHD reported mood or anxiety disorders (Horner et al., 2000; Bromberg et al., 2003; Kovacs et al., 2009b). In another study, 20% of ACHD reported symptoms consistent with posttraumatic stress disorder (Ferguson and Kovacs, 2016). The following factors have been associated with psychological distress in this population:

• Loneliness;
• Fear of negative evaluation;
• Imposed limits;
• Low capacity for physical exercise;
• Perceived health status (Callus et al., 2013).

When it comes to the neurocognitive functioning, there are indications that the manifestation of the pediatric-onset neurocognitive deficits may become more evident during adulthood and that cognitive functioning may be negatively affected by adult inset factors, such as heart failure and arrhythmias. In particular, during adulthood deficits in executive functioning have been reported (Daliento et al., 2005; Franklin et al., 2014).

In qualitative studies conducted on adults, it has been reported that having this condition can lead to feeling different, followed by attempts and struggle to feel normal and to be perceived as being normal by others (Gantt, 1992, 2002; Claessens et al., 2005; Berghammer et al., 2006; Verstappen et al., 2006).

Some examples of the experiences shared by this population include hospital visits, cardiac testing, being on medication, restrictions when it comes to school activities, interventions, and having a scar. Other challenges also depend on health status, and these could include absences at school or at university, which could lead to academic struggles, inability to participate in childhood activities which entail particular physical exertion.

Other possible consequences which have been reported are feelings of alienation, isolation, and marginalization, because of this condition often being invisible to others, dehumanization and disempowerment when the patients are reduced to their impairment and difficulties with care provision due to the confusing disparity between the patients’ experience of the illness and the picture described by the medical professionals in the past. All this can have an impact on body image, self-esteem and also can possibly lead to other psychological difficulties (Morton, 2011, 2012). Possible physiological variables linked to cardiac anomalies which could impact the disruption of connectedness and hinder the feeling of safety have been explored (Morton, 2017).

When it comes to the small minority of patients who experience on-going significant cyanosis, it was reported that they often suffered teasing, rejection, and being given distressing nicknames (Horner et al., 2000). Impediments in sexual life have also been reported among ACHD with implantable cardioverter-defibrillators; greater shock anxiety has been associated with poorer sexual functioning (Cook et al., 2013).

## Clinical Psychology Services for ACHD Patients

It has been reported by patients with CHD and their families that there is a need for psychosocial care (Kovacs et al., 2009a; Lesch et al., 2014; Levert et al., 2016). The main psychological concerns reported by ACHD patients in a recent study were generalized anxiety, health-/heart-related anxiety, depressed mood, and difficulty coping with a medical condition (Ferguson and Kovacs, 2016).

The necessity of adequate screening and identification of children with CHD when it comes to developmental, neuropsychological, and emotional problems has been recommended by the American Heart Association (Marino et al., 2012). It has also been reported that acting early can help to avoid the persistence of psychological problems later on in life (Hofstra et al., 2002).

In a recent perspective article on how psychological care can make a difference in congenital heart health (Kasparian et al., 2016), an integrated model of psychological care was presented. All health professionals involved in care are invited to assess the universal recognition of psychological needs. Health professionals with expertise in psychosocial training are invited to screen for psychological distress through validated screening tools. Specific assessment for psychological distress can be carried out by health professionals with expertise in psychosocial screening, by utilizing specific evidence based psychosocial interventions. Finally mental health specialists can provide diagnosis of psychopathology, by providing specialist psychological and psychiatric interventions, such as psychotherapy.

The necessity of including specialized mental health care for ACHD patients was reported in international working groups (Connelly et al., 1998; Warnes et al., 2001; Webb and Williams, 2001; Deanfield et al., 2003). Although at this time, screening for depression, anxiety, and other mood disorders is not in the ACHD guidelines, ACHD care providers are invited to be aware of these issues and intervene or refer when appropriate (Stout et al., 2015). Should there be an implementation of psychological screening programs, it is important to ensure that there are enough resources to score the chosen measure, that the results are discussed with the patients and that mental health care is provided to patients with both elevated distress and an interest in treatment (Lui et al., 2017). This is especially important because there seems to be under-diagnosis and under-treatment of psychosocial concern are present in ACHD patients (Bromberg et al., 2003); for this reason it is important to include a psychologist in ACHD teams, preferably ones with specialized cardiac experience (Kovacs et al., 2006).

Referral to a clinical psychologist could be indicated for the following:

• When the patients become aware they have the condition (especially if later in life) and if there are changes in the cardiac status;
• The transition from pediatric to adult care results to be difficult for these patients;
• Adjustment to cardiac devices and surgical preparation through relaxation and cognitive behavioral techniques;
• Maximization of adherence and behavioral modification through psychoeducation and motivational interviewing;
• Dealing with anticipatory grief and mortality;
• Psychosocial difficulties and problems with peers (Kovacs et al., 2006; Callus et al., 2015).

There should always be clear referral indications in the teams caring for ACHD (Kovacs et al., 2006). Referral to other specialists such as psychiatrists (Bassett et al., 2005) could also be necessary.

Up till 2013 there were no randomized controlled trials to investigate the efficacy of these interventions (Lane et al., 2013). There are indications that ACHD benefit from attending a brief course of psychotherapy (eight sessions) including cognitive therapy, relaxation training skills, and communication training skills in order to reduce psychological distress (Ferguson and Kovacs, 2016) and recently, the efficacy of psychosocial intervention, the Adult Congenital Heart Disease-Coping And REsilience (ACHD-CARE) is being evaluated (Kovacs et al., 2015).

Although the evidence for psychosocial interventions in the adult population is limited, successful programs have been reported in the literature when it comes to the pediatric population, in particular the Congenital Heart Intervention Program (CHIP), where it was demonstrated that there can be positive outcomes on maternal health and coping and infant cognitive development and feeding interactions, at least after a 6-month follow-up period (Doherty and McCusker, 2016). It is important also to consider these programs and also what effects they can have on a longer term.

## Patient Associations and Peer to Peer Programs

As was pointed out in the previous paragraphs, loneliness and feeling different is very pertinent in this population. For this reason associations can be very useful, because they give an occasion to the patients and the families to have a reference point and also to participate in aggregative activities (Campioni et al., 2015).

Specifically for CHD, the American Heart Association is running a program which is very well structured, in which there is a selection process and also the ones providing peer support are given supervision and indications on when a referral to a psychologist is necessary (ACHA, 2014).

When it comes to the selection of the patients for the program, they are requested to be in ACHD care for a minimum of 6 years, ideally have some previous voluntary experience, presenting three letters of recommendation one of which must be from an ACHD health care provider and another from an employer or volunteer supervisor or co-worker and to possess skills which are in line with the role. There is a 3-month trial period to determine if there is a good fit between the candidate and the association.

The candidates are requested to give a 1-year commitment to the program, with a time commitment of an average of 3–5 h per month. They are requested to attend an annual mandatory training program provided by ACHA and to attend monthly supervision calls and quarterly training webinars.

Currently there are some papers on peer to peer programs for patients with acquired heart disease (Hildingh and Fridlund, 2003; Arndt et al., 2009; Karwalajtys et al., 2009; Barg et al., 2012; Clark et al., 2012; Witt et al., 2016) with many studies involving patients with heart failure (Riegel and Carlson, 2004; Heisler et al., 2007, 2013; Mase et al., 2015). These programs should be the best comparison available for patients with CHD.

For the purpose of this paper, the study with a randomized controlled trial, a description of the training program and measure of efficacy will be briefly described (Heisler et al., 2013). The only thing specified regarding to the program is the patients randomized to the Reciprocal Peer Support received brief training in basic peer communication skills. No differences were verified from the nurse care management groups, when it comes to mean number of re-hospitalizations or death and there were no differences in improvements in 6-month measures of HF-specific quality of life or social support. When it comes to the program in itself, it was specified that the patients participating were encouraged to speak on a weekly basis. In a later article (Jonkman et al., 2016) it was reported that no specific program characteristics were consistently associated with better effects of self-management interventions in heart failure patients. It was, however, indicated that longer duration seemed to improve the effect of self-management interventions on several outcomes and that future research is necessary in this area.

## An Integration of the Clinical Psychology Service and Peer to Peer Support in Italy: A Practical Example in Italy

Following the guidelines mentioned previously (Connelly et al., 1998; Warnes et al., 2001; Webb and Williams, 2001; Deanfield et al., 2003; Stout et al., 2015; Lui et al., 2017) and the APPROACH study (Apers et al., 2015) a clinical psychology protocol has been created at the IRCCS Policlinico San Donato When it comes to the clinical activities in the operative unit in the hospital, the following activities are provided:

• Presence of the psychologist during the meetings of medical staff including, cardiac surgeons, pediatric and adult congenital cardiologists, anesthesiologist and during the weekly multidisciplinary psychosocial meeting;
• All ACHD patients who are hospitalized prior an intervention (both cardiac surgery and cardiac catheter) undergo a psychosocial evaluation through the administration of several questionnaires and a psychological session. These included acquiring data about their family and work situation, previous psychological and psychiatric visits, lifestyle, and the Hospital Anxiety and Depression Scale;
• Psychological interventions are provided according to the results of the evaluation and the indications of the medical, paramedical, and psychosocial staff
• All medical and paramedical staff are requested to alert the psychology service when the following are present:
  • Disturbances in mood, with particular attention to the presence of suicidal ideation
  • Anxiety disorders
  • Previous psychiatric diagnosis and intake of psychotropic drugs
  • Eating disorders abuse of alcohol and/or narcotic substances
  • Aggressive behavior (both self-injurious and to others), of patients and/or family members non-collaboration and non-compliance with medical care
  • Dissociative aspects and escape from reality
  • Neuropsychological disorders

Individual sessions of psychosocial support, stress reduction and relaxation and psychotherapy are provided accordingly and a referral to consultant psychiatrist is made when required. In particular, a psychiatrist is consulted in the case of psychotic hallucinations or delusions, psychiatric (co)morbidity and during end of life decisions.

Patients and their family members have the possibility to request individual and family clinical psychological sessions or psychotherapy, depending on the level of psychological distress manifested during hospitalization and also once they are discharged and during follow-up.

## Provision of Peer to Peer Support

The Association AICCA (Italian Congenital Heart Disease Organization) also operates within the IRCCS Policlinico San Donato, in the Department of Cardiology and Pediatric Cardiac Surgery and the Congenital Adult and Postoperative Intensive Care.

A peer counselor who also coordinates the activity of the association is also available for the patients and their family members. The association staff is present during the weekly multidisciplinary psychosocial meeting and the daily patient admission, to verify if there are any psychosocial issues. They provide assistance also when it comes to logistic, economic and bureaucracy issues, in relation to the condition. Social activities and outings are organized for the patients, their families, and their friends approximately once every 2/3 months.

## Discussion and Conclusion

In this perspective paper, it was highlighted that both clinical psychological services and peer to peer support are extremely important when it comes to the management of a chronic condition, with the specific example of ACHD patients. When possible, clinical psychologists with specific expertise and who are dedicated to these patients and their families should be present in the medical programs. An active collaboration with the local associations is also advised.

Since both kinds of services are currently available for the hospitalized patients in our center, one of the challenges which presented itself, was the fact that the medical professionals were sometimes confused as to who to refer the patients and their families to and for what problems. It was established that patients were primarily referred to the clinical psychology service when the levels of psychological distress were higher. It was also clarified that peer to peer support was more indicated, when it comes to loneliness and problems of a more social nature.

The benefits of this integrated system are that the patients who provide peer to peer support and also the association staff, are inserted in a network where they can receive supervision and also make a referral to a mental health specialist in case of increased psychological distress. The distinction and clarification of these two different kinds of services, reduces the risks of overburdening when it comes to peer to peer support. The medical staff is also instructed on which professional service to refer to for specific psychological, psychosocial, and psychiatric problems, increasing the possibility of providing effective mental health treatment and social support.

In addition to this, this example is an illustration of the importance of the collaboration between the clinical psychology service and the local associations (when both are available), in order to give indications for social and peer support when necessary and vice-versa, referral to psychology services when patients require it or when psychological distress is elevated.

Concluding, further studies are necessary in order to establish the effectiveness of both psychology services and peer to peer support programs for this population.

## Author Contributions

EC and GP both made substantial contributions in the conception, writing, and final approval of the article.

## Conflict of Interest Statement

The authors declare that the research was conducted in the absence of any commercial or financial relationships that could be construed as a potential conflict of interest.
